# Fluorescence confocal microscopy for evaluation of fresh surgical specimens and consecutive tumor cell isolation in rare pediatric tumors

**DOI:** 10.1007/s00428-024-03861-1

**Published:** 2024-07-09

**Authors:** S. Gretser, M. N. Kinzler, T. M. Theilen, P. J. Wild, M. Vogler, E. Gradhand

**Affiliations:** 1 Goethe University Frankfurt, University Hospital, Dr. Senckenberg Institute of Pathology, Theodor-Stern-Kai 6, 60590 Frankfurt Am Main, Germany; 2 Goethe University Frankfurt, University Hospital, Medical Clinic 1, Frankfurt Am Main, Germany; 3 Goethe University Frankfurt, University Hospital, Department of Pediatric Surgery and Pediatric Urology, Frankfurt Am Main, Germany; 4 Goethe University Frankfurt, Institute for Experimental Pediatric Hematology and Oncology, Frankfurt Am Main, Germany

**Keywords:** Fluorescence confocal microscopy, Digital pathology, Pediatric pathology, Pediatric surgery

## Abstract

Fluorescence confocal microscopy (FCM) is an optical technique that uses laser light sources of different wavelengths to generate real-time images of fresh, unfixed tissue specimens. FCM allows histological evaluation of fresh tissue samples without the associated cryo artifacts after frozen sectioning. The aim of this study was to prospectively evaluate pediatric tumor specimens and assess their suitability for fresh tumor sampling. In addition, we aimed to determine whether tumor cell isolation for stable cell culture is still feasible after FCM imaging. Pediatric tumor specimens were imaged using FCM. Tumor viability and suitability for tissue sampling were evaluated and compared with H&E staining after paraffin embedding. In addition, FCM-processed and non-FCM-processed tissue samples were sent for tumor cell isolation to evaluate possible effects after FCM processing. When comparing estimated tumor cell viability using FCM and H&E, we found good to excellent correlating estimates (intraclass correlation coefficient = 0.891, *p* < 0.001), as well as substantial agreement in whether the tissue appeared adequate for fresh tissue collection (*κ* = 0.762, *p* < 0.001). After FCM, seven out of eight samples yielded passable cell cultures, compared to eight out of eight for non-FCM processed samples. Our study suggests that the use of FCM in tumor sampling can increase the yield of suitable fresh tumor samples by identifying viable tumor areas and ensuring that sufficient tissue remains for diagnosis. Our study also provides first evidence that the isolation and growth of tumor cells in culture are not compromised by the FCM technique.

**Supplementary Information:**

The online version contains supplementary material available at 10.1007/s00428-024-03861-1.

## Introduction

In general, cancer is rare in pediatric patients, and the global incidence is poorly quantified [1]. Among all cancer patients, pediatric cancer patients account for only about 1%. In the USA, the incidence of childhood cancer in 2015–2019 was 173 per million population [2]. Due to the low incidence and high heterogeneity of pediatric tumors, translational research is hampered by small patient populations and sparsity of available tissue. Biobank collaborations are working together to maximize the scarce supply of fresh biological tissue samples [3]. In addition, large pediatric oncology study groups, such as the Children’s Oncology Group (COG) in the USA or the International Society of Pediatric Oncology (SIOP) in Europe, search for potential molecular targets in pediatric cancer requiring representative and viable fresh tumor tissue [4, 5]. Fresh tissue samples are obtained either by tissue biopsy or as part of the resection specimen. In both cases, representative vital tumor areas must be identified macroscopically. Histologic confirmation of the adequacy of the tissue is usually not performed, as a required frozen section would inhibit the further use of the tissue. For larger resection specimens, formalin-fixed, paraffin-embedded (FFPE) mirror images of the freshly removed tissue are taken, confirming the viability of the freshly removed tissue retrospectively. One solution to this problem is ex vivo fluorescence confocal microscopy (FCM), which allows immediate evaluation of tissue adequacy.

FCM is an optical technique that uses laser light sources of different wavelengths to generate real-time images of fresh, unfixed tissue samples. To date, FCM has been best established in dermatopathology, but has also been studied in many other human surgical tissues such as breast, genitourinary, and gastrointestinal [6]. Recently, FCM has been extended to applications in which flexible alternatives to conventional frozen section are needed, such as in liver transplantation [7]. In pediatric tumor specimens, however, the applicability of FCM has not been investigated.

## Methods

### Cohort and study design

Diagnostic tumor resection specimens of pediatric patients submitted by the Department of Pediatric Surgery and Urology at the University Hospital Frankfurt, Germany, between January 2023 and December 2023 were included in this study. From each specimen, a representative section was taken for FCM imaging, followed by regular paraffin embedding. In the case of heterogeneous tumors, multiple sections were taken. Tumor vitality and adequacy for tissue sampling were blindly assessed by a pediatric pathologist first on the FCM images and later on the corresponding H&E slides. Tumor vitality was estimated as the percentage of vital tumor cells on the tissue surface. Adequacy of tissue sampling was assessed by taking viability, cellularity, and vital non-tumorous tissue into account. When possible, another section was imaged by FCM and submitted for tumor cell isolation, followed by an adjacent tumor section to isolate cells without FCM procession for comparison. The study protocol was approved by the local ethics committee of the University of Frankfurt (project number: UCT-53–2022/SPO-1–2022).

### FCM imaging

FCM image acquisition was performed with the VivaScope 2500 M-G4 (MAVIG GmbH, VivaScope Systems, Munich, Germany). For image acquisition, the tumor specimen was pretreated with 70% ethanol for 10 s, followed by a wash step with 0.9% physiological saline solution for 10 s. The specimen was then stained with the fluorescent dye acridine orange (0.6 mM; Sigma-Aldrich, St. Louis, MO, USA) for 30 s, followed by another washing step. The specimen was then mounted on a glass slide and fixed with small sponges and another glass slide. After imaging, the specimens were fixed in 4% PBS-buffered formaldehyde immediately after scanning for further histologic processing. Specimens submitted for tumor cell isolation were processed in Dulbecco’s modified Eagle’s medium (DMEM, Gibco) with 10% fetal bovine serum (Biochrom). In parallel, unprocessed counterparts of the same tumor region were also submitted for tumor cell isolation as a control group. To ensure that the submitted samples were taken from viable parts of the tumor, representative, mirroring blocks from the sampling sites were taken for histological examination. This also enabled us to ascertain which cells were to be isolated in the case of a heterogeneous tumor (e.g., nephroblastomas).

### Tumor cell isolation

For isolation of tumor cells, tissues were placed in a petri dish in a sterile environment under a safety hood. Cells were isolated by cutting of the tissue into smaller pieces followed by squeezing of the tissues through a filter mesh. Cells were washed in RPMI1640 medium (Gibco) by centrifugation at 1400 rpm for 5 min. The pellet was resuspended in culture medium containing DMEM/F12 (Gibco) with 2% B27 (Thermo Scientific), 1% N2 (Thermo Scientific), 1% Pen/Step (Gibco), 0.5% Gentamicin (Sigma), 0.5% Amphotericin (Thermo Scientific), 1% Non-Essential Amino Acids (Gibco), 1% Sodium Pyruvate (Gibco), 1.25 mM N-acetylcysteine (Sigma), 1 U/ml heparin (Sigma), 20 ng/ml hEGF (Peprotech), 40 ng/ml hFGF-basic (Peprotech), 20 ng/ml hIGF1 (Peprotech), 10 μM Y-27632 (AbMole), and 5 μM A83-01 (Tocris) and cultured in cell culture flasks. Pictures of growing cultures were taken at Olympus IX71 with CellSens Standard software (Olympus). To ascertain that the isolated cells were indeed tumor cells and not cells from the tumor microenvironment (e.g., fibroblasts), the isolated cell lines were stained for tumor-associated surface markers such as CD56, which distinguishes several pediatric tumors from fibroblasts [8].

### Statistical analysis

Intraclass-correlation (two-way mixed effects, consistency, single measurement, ICC3,1) was used to measure the reproducibility of the estimated vitality of FCM and conventional H&E staining. Agreement/correlation was interpreted according to Koo and Li (intraclass correlation coefficient < 0.5 = poor, 0.51–0.75 = moderate, 0.76–0.9 = good, > 0.9 = excellent) [9]. Reproducibility of tissue adequacy for FCM compared to conventional H&E staining was statistically analyzed using Cohen’s kappa (*κ*). Agreement was interpreted according to Landis and Koch (*κ* < 0.00: poor, *κ* = 0.00–0.20: slight, *κ* = 0.21–0.40: fair, *κ* = 0.41–0.60: moderate, *κ* = 0.61–0.80: substantial, and *κ* = 0.81–1.00: almost perfect) [10]. All data were analyzed with SPSS 29 (IBM) statistical software.

## Results

Thirteen tumor specimens from 11 patients were included in this study. Approximately one-third of the cases were diagnosed as nephroblastoma (38.5%), while the remaining cases consisted of other pediatric tumor entities (Table 1). Of the 13 tumors, nine received neoadjuvant therapy prior to surgical removal. Because of the heterogeneous nature of the tumors, a total of 20 FCM slides with corresponding H&E histology were prepared from these 13 specimens for further analysis.

**Table 1:** Patient cohort

	Patients, *n* = 11 (%)
Mean age, years (range)	8.46 (1–18)
Sex	
Male (%)	6 (54.5)
Female (%)	5 (45.5)
Diagnosis	Specimen, *n* = 13 (%)
NB (%)	5 (38.5)
ERMS (%)	2 (15.3)
NeuB (%)	1 (7.7)
SS (%)	1 (7.7)
RCC (%)	1 (7.7)
OS (%)	1 (7.7)
YST (%)	1 (7.7)
LGST (%)	1 (7.7)

### Tumor vitality and tissue adequacy

**Fig. 1:**
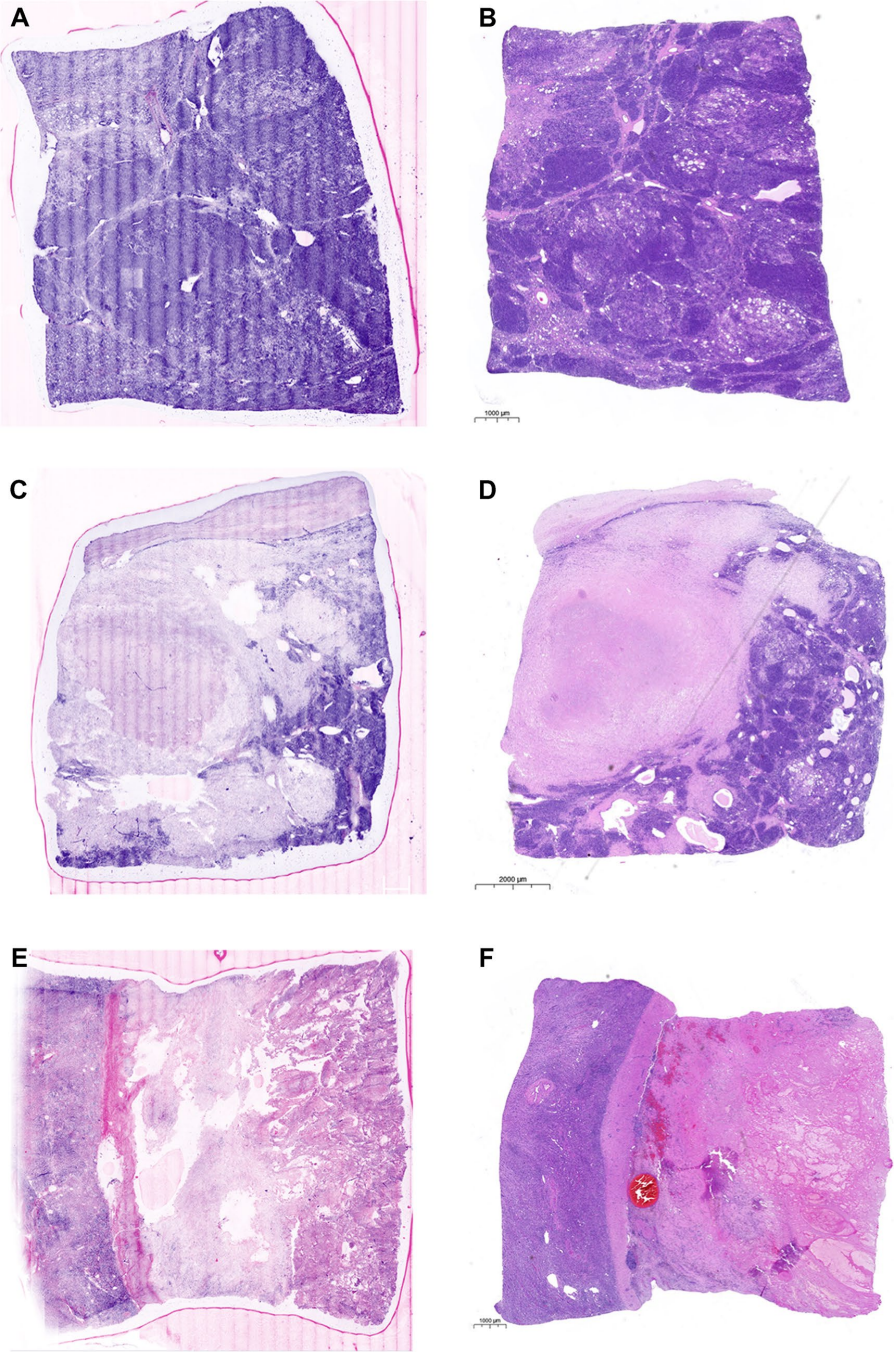
Representative FCM images **(A, C, E)** with their respective H&E counterpart **(B, D, F). A** and **B** illustrate fully viable nephroblastoma, **C** and **D** depict partially viable nephroblastoma (necrosis in the upper left), and **E** and **F** show a fully necrotic nephroblastoma (necrosis on the right, viable kidney tissue on the left)

**Fig. 2:**
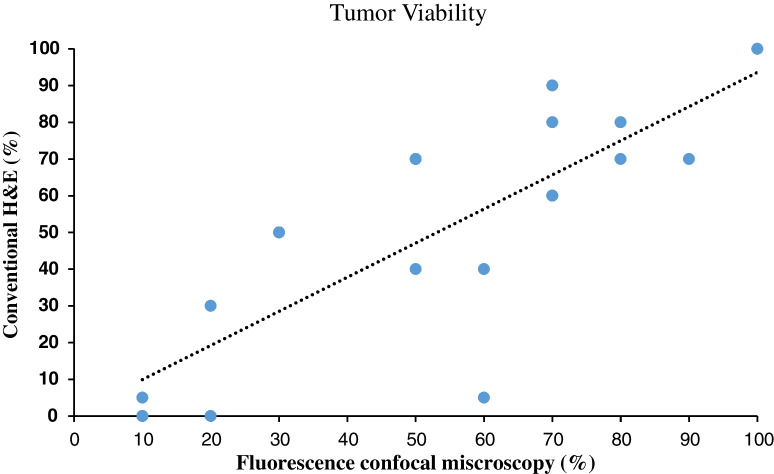
Scatter plot for tumor viability estimated on conventional H&E stains and FCM showing good to excellent correlation between FCM and H&E (intraclass correlation coefficient = 0.891, *p* < 0.001)

**Fig. 3:**
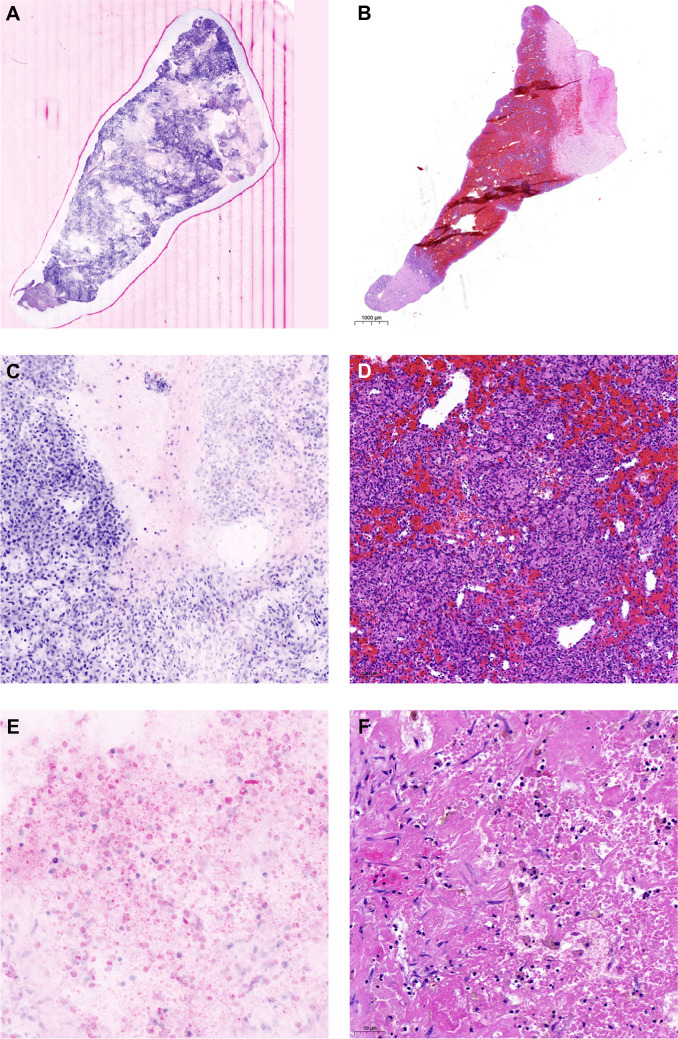
Potential pitfalls in FCM image interpretation**. A** and **B** depict the same specimen from a renal clear cell carcinoma. **B** illustrates the H&E staining of the sample following fixation and sectioning, while **A** depicts the corresponding FCM image. A notable decline in vital tumor cells can be observed in the H&E-stained slide due to the different levels of the tumor sample. **C** and **D** illustrate the same area of a renal clear cell carcinoma. The artificially bleached cells in the FCM image (**C**) can be mistaken for non-viable cells, whereas they are fully viable on the H&E image (**D**), leading to a lower overall estimate of tumor viability in FCM (**D**). **E** and **F** show necrosis in a nephroblastoma in FCM (**F**) and H&E (**E**), in which some of the scattered lymphocytes may be mistaken for vital cells in the FCM image

As a first step, we wanted to see if tumor vitality could be assessed by FCM. In general, we observed that vital and avital tumor cell areas could be well identified by FCM (Fig. 1). Regions of tumor regression following neoadjuvant therapy, including fibrosis and necrosis, were also classified as non-viable. When comparing the estimated tumor cell vitality by FCM and H&E, we found good to excellent correlating estimates (intraclass correlation coefficient = 0.891, *p* < 0.001) with an average discrepancy of 15% (95% CI = 11.05–19.95) as seen in Fig. 2 (Fig. 2). For the estimation of tissue adequacy, both FCM and H&E slides were assessed independently, taking into account viability, cellularity, and vital non-tumorous tissue. Substantial agreement was observed as to whether the tissue was adequate for fresh tissue collection (agreement in 18 out of 20 evaluated slides, *κ* = 0.762, *p* < 0.001). As a next step, we wanted to identify possible factors for the discrepancy in estimated tumor cell vitality between FCM and H&E. One factor, which we were able to identify, is a purely methodological problem. While the FCM image was taken directly from the surface of the specimen, the specimen had to be paraffin-embedded and sectioned to generate the slide. In the end, different planes of the specimen were evaluated, which accounts for some of the discrepancy in estimated tumor viability (Fig. 3A–B). The case shown in Fig. 3A–B is the case with the largest discrepancy in estimated tumor cell viability (60% in FCM vs. 5% in H&E). As can be clearly seen in the images, the proportion of vital tumor cells is significantly lower in the H&E stained slide, highlighting the problem of different sectioning levels in the compared samples. Another factor we could identify is that in the case of an uneven section surface, some nuclei would fade out due to this uneven surface, giving the impression of non-viable tumor cells, leading to a lower estimation of viable cells in the FCM image (Fig. 3C–D). In addition, some non-viable tumor cells with intermixed lymphocytes can sometimes be misinterpreted as viable tumor cells, resulting in slightly higher estimation in the FCM image (Fig. 3E–F). To ensure that there were no effects on ancillary tests such as immunohistochemistry, we performed immunohistochemistry on tissue with and without FCM-processing. Our results showed that there were no differences between the two groups (Supplementary Fig. 1).

### Tumor cell isolation after FCM

**Fig. 4:**
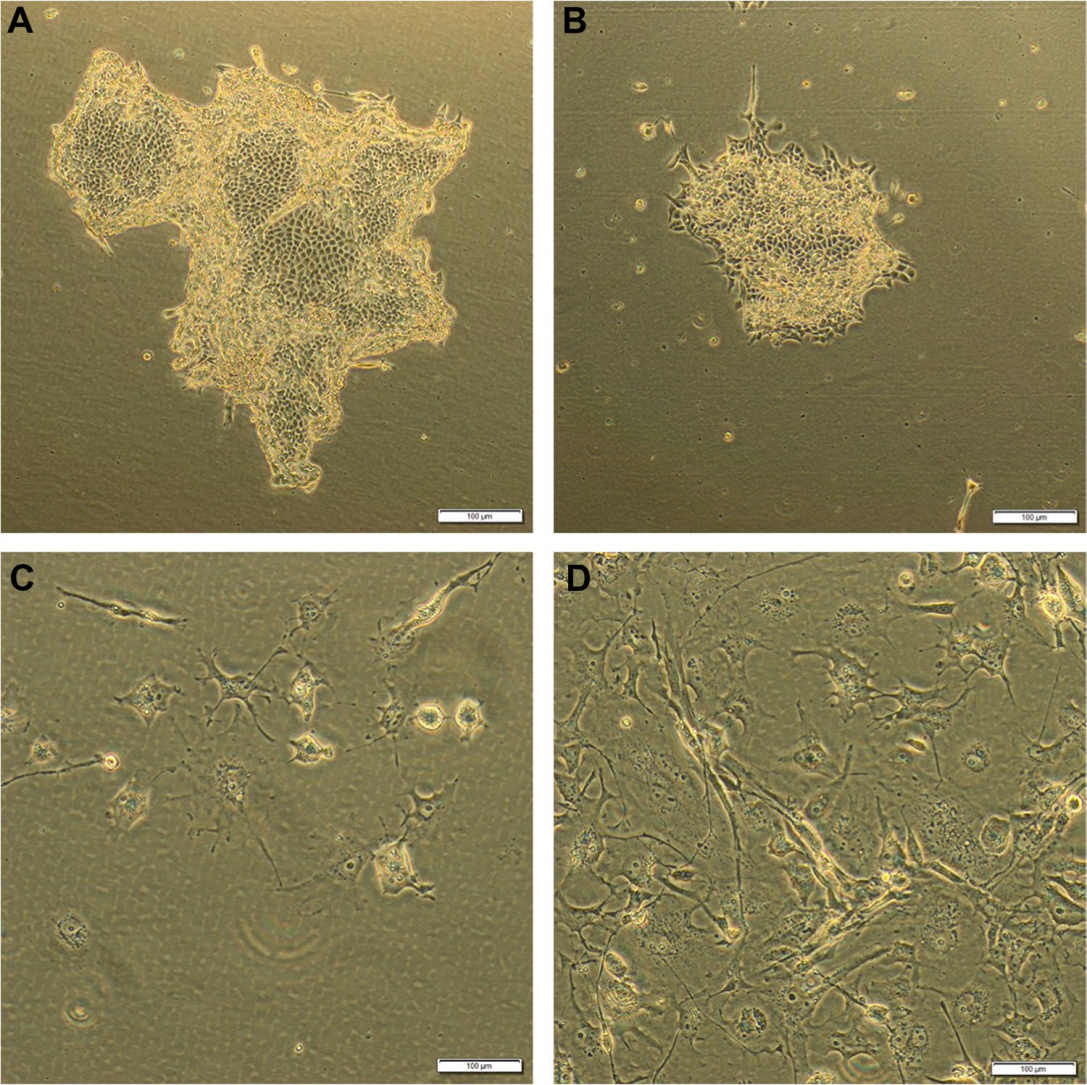
Sample images of isolated cultured cells. Images of isolated cell lines from a renal clear cell carcinoma (**A**, **B**) and an embryonal rhabdomyosarcoma (**C**, **D**) are presented. Following FCM-processing (**A**, **C**) or without FCM-processing (**B**, **D**), the isolated cell lines demonstrated no obvious morphological differences with solid growth in the carcinoma and discohesive growth in the sarcoma (magnification 40 ×)

The second focus of this study was to determine whether FCM-treated material was still suitable for tumor cell isolation for cell culture. In eight of the 13 tumor samples (62%), tissue for tumor cell isolation could be obtained with and without prior processing for FCM. Passagable cell cultures were obtained from all eight tissue samples that were not examined using the FCM and therefore did not receive the corresponding pretreatment. After FCM and its pretreatment conditions, seven of the eight samples yielded passagable cell cultures (Fig. 4). Of these seven, one had fungal contamination and had to be discarded.

## Discussion

Adequate tumor sampling is essential for both diagnostic and scientific purposes. In the diagnostic process, tumor specimens are formalin-fixed, and representative tumor specimens are paraffin-embedded. Typically, multiple tumor samples are processed to account for tumor heterogeneity and to identify vital tumor areas in macroscopically non-uniform tumors. From these paraffin-embedded samples, the most appropriate area can be identified and used for post-diagnostic research purposes. Many methods, such as most sequencing-based techniques, can be performed on paraffin-embedded tissue [11], but there are some discrepancies compared to fresh snap-frozen tissue [12, 13]. For other methods, such as tumor cell isolation, adequate fresh tumor tissue is mandatory. Although in some cases vital tumor areas can be identified macroscopically, in other cases the identification of adequate areas can be challenging. Prospective studies aimed at identifying potential therapeutic targets also rely on viable, representative tumor samples [4, 5]. In these cases, histological confirmation of the adequacy of the tumor sample would be very useful, but current techniques such as frozen sectioning render the material useless for cell isolation, waste some of the tumor material, and create cryo artifacts. FCM could be of great help to solve this problem, but so far FCM has not been studied on pediatric tumor samples. The FCM technique has several major advantages over conventional frozen sectioning. First of all, the operation of the microscope can be performed after a short induction training. This eliminates the need for an entire frozen section laboratory with an on-call technician and allows for more flexible examination of tissues. Another major advantage is that FCM does not require tissue to be frozen and sectioned. Loss of tissue due to cutting and impaired immunoreactivity due to freezing can be avoided. Both immunohistochemistry and molecular analysis can still be performed after FCM, making it the optimal tool for tumor sampling [14].

Until now, it has not been investigated whether tumor cells can still be isolated for ex vivo cell culture after tissue processing with FCM. The aim of this study was to prospectively investigate pediatric tumor specimens and to evaluate the adequacy of pediatric tumor specimens for fresh tumor sampling.

In this study, we were able to demonstrate that FCM imaging enables the user to reliably identify representative tumor areas for fresh tumor sampling when compared to corresponding H&E specimens as a gold standard. In addition, tumor viability could be accurately assessed in most cases. However, the procedure has some limitations. We were able to identify some of the factors that cause the discrepancy between FCM and H&E, such as different sectioning planes, staining, and interpretation errors. A final factor, which needs to be addressed, is the fact that the FCM technique is not routinely performed in most pathology departments. Therefore, the experience of the pathologist in handling the microscope and interpreting the images is still a matter of practice and may explain some of the discordance. However, these limitations to the current study can be expected to be minimized with prolonged implementation and experience in the FCM technique.

While it has been shown that most downstream methods are still possible after FCM [14], it has not been shown whether cell isolation for cell culture is affected by the FCM process. In this study, we were able to show that cell isolation and subsequent cell culture growth is not affected by the staining or imaging process of FCM. The main problem we encountered with cell isolation was that the size of the sample processed by FCM was limited by the size of the glass slide used for FCM. This resulted in smaller tumor samples after FCM processing compared to their non-FCM-processed counterparts. This problem is solvable by submitting more tissue from the FCM examined area. In addition, FCM processing requires several steps under non-sterile conditions, which may explain the one case of fungal contamination we experienced in our cell isolation experiments. Stricter sterile working conditions may prevent possible contamination. Alternatively, FCM could be used only to identify suitable areas in larger tumors, and then fresh tissue samples could be sent for cell isolation.

In this study, we focused on the investigation and assessment of tumor vitality and quality control of fresh tumor sampling in large tumor resection specimens. In most of these cases, the diagnosis had already been made on biopsy material. Our results on these large specimens encourage the investigation of the practicability of FCM in diagnostic biopsies. The quality control of these biopsies as well as the preliminary diagnosis could be performed immediately after the biopsies are taken to ensure the adequacy of the biopsy and to accelerate the diagnostic process.

## Conclusion

Our study was able to demonstrate that the use of FCM for pediatric tumor samples could increase the yield of fresh tumor samples in this particularly small tissue sample cohort. Furthermore, we are the first to show that the isolation of tumor cells is not affected by the FCM technique.

## Supplementary Information

Below is the link to the electronic supplementary material.

Supplementary Fig. 1 Representative immunohistochemistry stains after FCM-processing (A, C, E) and without FCM-processing (B, D, F). There is no discernible difference in the immunohistochemical staining pattern after FCM-processing when compared to non-FCM-processed parts of the same tumor. Figures A & B show stainings for WT1 in a Nephroblastoma, Figures C & D show stainings for MyoD1 in an embryonal Rhabdomyosarcoma and Figures E & F show stainings for CD56 in a Neuroblastoma (PNG 5001 KB)

## Data Availability

Imaging data are available from the corresponding author on reasonable request.

## Abbreviations

*NB*: Nephroblastoma
*ERMS*: Embryonal rhabdomyosarcoma
*NeuB*: Neuroblastoma
*SS*: Synovial sarcoma
*RCC*: Renal clear cell carcinoma
*OS*: Osteosarcoma
*YST*: Yolk-sac tumor
*LGST*: Low-grade spindle cell tumor with TRIM::BRAF
